# A novel class of tsRNA signatures as biomarkers for diagnosis and prognosis of pancreatic cancer

**DOI:** 10.1186/s12943-021-01389-5

**Published:** 2021-07-17

**Authors:** Fangfang Jin, Liuqing Yang, Weixiang Wang, Na Yuan, Shoubin Zhan, Ping Yang, Xi Chen, Tonghui Ma, Yanbo Wang

**Affiliations:** 1 School of Medicine & Holistic Integrative Medicine, Nanjing University of Chinese Medicine, Nanjing, 210023 China; 2 Department of Infectious Diseases, Department of Gastrointestinal Surgery, The First People’s Hospital of Lianyungang, The First Affiliated Hospital of Kangda College of Nanjing Medical University, Lianyungang, Jiangsu China; 3 Nanjing Drum Tower Hospital Center of Molecular Diagnostic and Therapy, Chemistry and Biomedicine Innovation Center (ChemBIC), State Key Laboratory of Pharmaceutical Biotechnology and Department of Physiology, Jiangsu Engineering Research Center for MicroRNA Biology and Biotechnology, NJU Advanced Institute of Life Sciences (NAILS), School of Life Sciences, Nanjing University, Nanjing, 210023 China; 4 Department of Clinical Laboratory, The Affiliated Drum Tower Hospital of Nanjing University Medical School, Nanjing, 210093 China

**Keywords:** tsRNA, Biomarker, Pancreatic cancer

## Main text

Pancreatic cancer (PC) is the third leading cause of cancer-related mortality worldwide and most PC patients are advanced once diagnosed [1]. Identification of novel biomarkers with high sensitivity and specificity for early diagnosis and prognosis is currently considered the best strategy to improve PC therapeutic effects.

tRNA-derived small RNAs (tsRNAs), 18 ~ 40 nt in length, are novel small non-coding RNAs generated from precursor or mature tRNAs [2]. Recent evidences indicated dysregulation of tsRNAs in various cancers [3–6], suggesting that tsRNAs may play vital roles in tumorigenesis. The potential use of circulating nucleic acids in serum and other body fluids for cancer screening and prognosis has recently emerged [7–9]. tsRNAs have also been found stable in circulation and present in a surprisingly higher percentage than miRNAs [10]. However, the diagnostic values and biological functions of tsRNAs in serum, especially for PC, are still ambiguous and intriguing.

In this study, we tested the hypothesis that there is a tsRNA profile that can be used as fingerprint to diagnose in early stage and/or predict clinical outcome of PC. We used RNA sequencing, qRT-PCR and in situ hybridization to identify PC-associated tsRNA signatures in human sera, tissues, cancer cells and mouse model. The altered tsRNAs profiles in serum and cancer tissue showed great promise as novel biomarkers for early diagnosis and prognosis of PC.

## Results and discussion

### Ectopic tsRNA signatures in pancreatic cancer serum

**Fig. 1:**
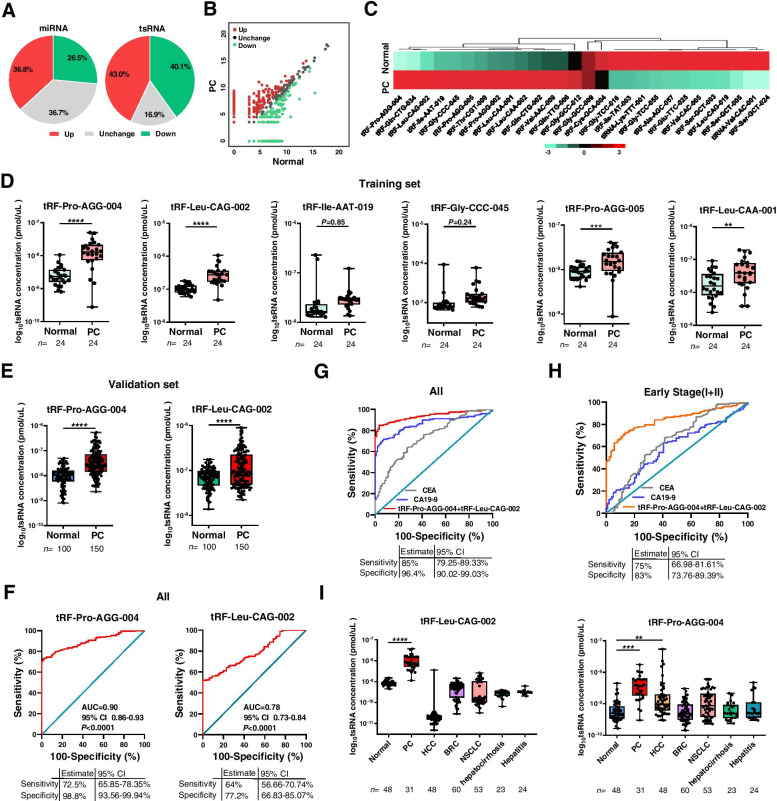
Identification of novel tsRNA biomarkers from serum samples of PC patients. **A** Proportion of changed miRNAs and tsRNAs in serum from PC patients compared with normal controls. **B** The scatter plot figuratively expresses the changes in tsRNAs expression. **C** Hierarchical clustering indicates the differences in tsRNA expression profiling between two groups. **D** The six serum tsRNAs expression in PC patients in the training set. Serum samples from 24 PC patients and 24 controls were collected and subjected to qRT-PCR absolute quantification. **E** qRT-PCR shows the concentrations of tRF-Pro-AGG-004 and tRF-Leu-CAG-002 in 150 PC patients and 100 control individuals enrolled in the validation set. **F** ROC curves of tRF-Pro-AGG-004 and tRF-Leu-CAG-002 concentrations in serum samples from PC patients versus healthy controls. **G** Comparison of the diagnostic values of 2-tsRNA signature with CA19-9 and CEA in pancreatic cancer. **H** ROC analysis estimates the diagnostic value of 2-tsRNA signature in early stage pancreatic cancer. **I** qRT-PCR shows the serum concentrations of tRF-Pro-AGG-004 and tRF-Leu-CAG-002 in other types of diseases [including 31 PC patients, 48 HCC patients, 60 breast cancer (BRC) patients, 53 non-small cell lung cancer patients (NSCLC), 23 hepatocirrhosis patients, 24 hepatitis patients and 48 control individuals]. **P < 0.01, ***P < 0.001, ****P < 0.0001

To explore whether serum tsRNAs could serve as novel biomarkers for PC, we performed preliminary screening by small RNA sequencing followed by qRT-PCR validation on individual basis. First, RNAs were extracted from serum pools of 30 PC patients or 30 healthy controls (all samples were collected from The First People’s Hospital of Lianyungang) for sequencing. Length analysis showed that RNA from two groups exhibited different distributions (Fig. S1A). Among 433 tsRNAs and 1121 miRNAs detected, larger variations were found in tsRNAs than miRNAs in PC group compared with healthy controls (Fig. 1A, B), suggesting that tsRNAs may be more sensitive biomarkers for PC detection. The analysis resulted in 26 significant differentially expressed tsRNAs [CPM (counts per million) > 10 and fold-change > 10] in PC serum compared with healthy controls (Fig. 1C, Table S1).

Next, the findings were validated using an independent training cohort (24 PC patients, 24 healthy controls from The First People’s Hospital of Lianyungang). The probe-based RT-qPCR assay was reliable and reproducible for detecting tsRNAs as indicated by the linear standard curve from 10 fmol/L to 10 nmol/L for different concentrations of synthetic tsRNA standards (Fig. S1B). Absolute quantitation and relative comparison were used to validate the differential expression of six candidates from the top-ten upregulated tsRNAs in individual serum sample of 24 healthy controls and 24 PC patients (due to the length limitation, only six tsRNAs can be assessed by probe-based RT-qPCR). Absolute quantification identified 4 out of the 6 tsRNAs that were significantly elevated in PC serum compared to healthy controls (Fig. 1D). Relative comparison analysis (Plant-derived MIR2911 and *Caenorhabditis elegan-* derived cel-miR-39 serve as exogenous reference genes) showed that 2 out of the 6 tsRNAs (tRF-Pro-AGG-004, tRF-Leu-CAG-002) were signifcantly increased in PC patients (Fig. S2). Then we evaluated the effects of the two tsRNAs on risk score prediction, ROC analysis showed that tRF-Pro-AGG-004 and tRF-Leu-CAG-002 have higher AUC (0.88, 95% CI 0.77–0.99; 0.93, 95% CI 0.85–1.00) (Fig. S3). Altogether, tRF-Pro-AGG-004 and tRF-Leu-CAG-002 emerge as candidates for PC detection.

### Diagnostic value of differentially expressed serum tsRNAs in pancreatic cancer

Next, the expression of the two tsRNAs were further analyzed in a larger cohort of serum samples from two hospitals (validation set: 100 healthy controls *vs* 150 PC patients, including 46 PC patients *vs* 26 healthy controls from The First People’s Hospital of Lianyungang and 104 PC patients *vs* 74 healthy controls from The Affiliated Drum Tower Hospital of Nanjing University Medical School). Consistent with training set, the two tsRNAs were remarkably elevated in PC serum compared to controls (Fig. 1E). ROC analysis was performed to determine the diagnostic value of the 2-tsRNAs profile (in a total of 204 PC serum samples including the discovery set, training set and validation set). The two tsRNAs individually showed high diagnostic value with AUC (tRF-Pro-AGG-004: 0.90; tRF-Leu-CAG-002: 0.78) and performance (tRF-Pro-AGG-004: sensitivity 72.5%, specificity 98.8%; tRF-Leu-CAG-002: sensitivity 64%, specificity 77.2%) (Fig. 1F). In the combined analysis, the signature with 2 tsRNAs has higher AUC (0.94, 95% CI 0.91–0.96) and higher performance (sensitivity 85%, specificity 96.4%) compared with any of the single tsRNA (Fig. 1G). We further compared the diagnostic effects of the 2-tsRNAs signature to clinical PC biomarkers CA19-9 and CEA. While CA19-9 and CEA levels were elevated in PC serum (Fig. S4), ROC analysis showed that the 2-tsRNAs signature demonstrated superior diagnostic potential than CA19-9 and CEA (Fig. 1G). Intriguingly, the 2-tsRNAs combination demonstrated a significantly superior diagnostic accuracy for early PC patients (stage I and II) with an AUC of 0.84 exhibiting a sensitivity of 75.0% and specificity of 83.0% (Fig. 1H), better than CA19-9 (sensitivity 63.5%, specificity 59.6%) and CEA (sensitivity 63.1%, specificity 58.5%). Besides, detection of tRF-Pro-AGG-004 and tRF-Leu-CAG-002 expression in other types of diseases (including HCC, BRC, NSCLC, etc.) showed that the combined 2-tsRNAs signature had certain disease specific in PC (Fig. 1I, Table S3). These results demonstrated that serum tRF-Pro-AGG-004 and tRF-Leu-CAG-002 could be used as novel promising biomarkers for PC diagnosis, even in early stage.

### Elevated serum tRF-Pro-AGG-004 and tRF-Leu-CAG-002 originate from tumor cells

**Fig. 2:**
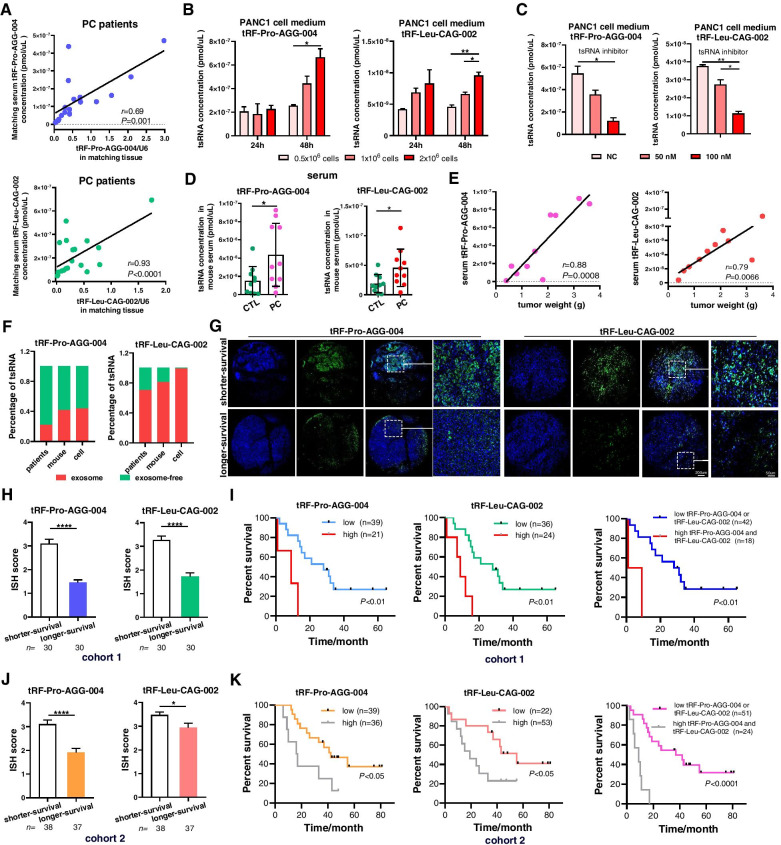
Elevated tsRNAsin tumor tissues as independent indicator for predicting survival time of PC patients. **A** tRF-Pro-AGG-004 and tRF-Leu-CAG-002 expression in PC tissues and paired serum from the same patients (*n* = 20). **B** The concentrations of tRF-Pro-AGG-004 and tRF-Leu-CAG-002 secreted in the PANC1 culture medium depend on cell number and duration of culture. **C** PANC1 cells were treatmented with tRF-Pro-AGG-004 or tRF-Leu-CAG-002 inhibitors, the concentrations of tRF-Pro-AGG-004 and tRF-Leu-CAG-002 secreted in the PANC1 culture medium were measured. **D** Serum tRF-Pro-AGG-004 and tRF-Leu-CAG-002 expression levels in normal mice and PC orthotopic transplantation tumor mice. **E** Significant correlation between serum tRF-Pro-AGG-004 and tRF-Leu-CAG-002 expression and tumor weight in mice. **F** tRF-Pro-AGG-004 in patient serum, mouse serum and cell medium is mainly in exosome-free fraction, whereas tRF-Leu-CAG-002 is mainly in exosome. The 60 PC patients in cohort 1 were divided into two groups: shorter-survival group (*n* = 30) and longer survival group (*n* = 30). The 75 PC patients in cohort 2 were divided into two groups: shorter-survival group (*n* = 38) and longer survival group (*n* = 37). **G** Representative results for in situ hybridization (ISH) staining of tRF-Pro-AGG-004 or tRF-Leu-CAG-002 in pancreatic cancer (PC) tissues from two groups. **H**, **J** ISH scores of tRF-Pro-AGG-004 and tRF-Leu-CAG-002 in two groups from two cohorts (according to positive rate of ISH staining, each sample was scored as 1(0–25%), 2(26–50%), 3(51–75%), 4(76–100%)). **I**, **K** Kaplan–Meier overall survival curve of PC patients in two cohorts based on ISH scores of tRF-Pro-AGG-004 and tRF-Leu-CAG-002, and combined tRF-Pro-AGG-004 and tRF-Leu-CAG-002. The optimal cut-offs of ISH was set (ISH score ≥ 3 as high and ISH score < 3 as low). *P < 0.05; **P < 0.01; ****P < 0.0001

To determine whether the high levels of the two tsRNAs in PC serum were induced by perturbations in PC tissues, we first examined the tissue and paired serum levels of tRF-Pro-AGG-004 and tRF-Leu-CAG-002 from the same patients in a independent clinical cohort (*n* = 20, Table S4) and observed an positive correlation (Fig. 2A). To further explore whether the increased tRF-Pro-AGG-004 and tRF-Leu-CAG-002 are released from PC cells, tsRNA expression was investigated in PANC1 culture medium over various time periods. We found that their expression were directly proportional to cell number and culture time (Fig. 2B). Moreover, treatment by two tsRNAs inhibitors in cells showed decreased tsRNAs levels in culture medium (Fig. 2C), suggesting that tsRNAs in culture medium were directly released from PC cells. We further confirmed above results in PC orthotopic transplantation tumor model. PAN02 cells were injected into mouse pancreas to establish a PC orthotopic transplantation tumor model (Fig. S5A,B). Quantification of tRF-Pro-AGG-004 and tRF-Leu-CAG-002 indicated significantly elevated expression in tumor tissue and serum specimens from PC-bearing mice compared with control mice (Fig. 2D, Fig. S5C). Furthermore, significant positive correlation was recognized between serum levels of two tsRNAs and tumor weights (Fig. 2E). Taken together, these results indicate that increased serum tRF-Pro-AGG-004 and tRF-Leu-CAG-002 were originated from tumor tissues.

We also checked the existence forms of circulating tsRNAs, and total RNA was isolated from exosomes and exosome-free supernatants from PC patient serum, PC mouse serum and PANC1 cell medium, respectively. Interestingly, the results showed that tRF-Pro-AGG-004 was highly enriched in exosome-free supernatants, whereas tRF-Leu-CAG-002 was preferentially existed in exosomes (Fig. 2F). The variable forms of two circulating tsRNAs indicate their different action and systemic regulatory mechanisms.

### Prognostic potential of the 2-tsRNAs signature in tumor tissues for PC patients

Tumor tissues may provide more information on tumor staging and prognosis. Traditional pathological staging systems may have reached their limit for predicting outcomes, and new molecular biomarkers may add values. Therefore, we checked and scored the two-tsRNAs expression in 2 clinical PC cohorts by ISH analysis in standard process. The results showed that tRF-Pro-AGG-004 and tRF-Leu-CAG-002 were significantly upregulated in PC tissues than paired normal tissues (Fig. S6, Tables S5 and S6). When we divided patients into shorter-survival group and longer-survival group according to the survival time, both tsRNAs were markedly higher in shorter-survival group (Fig. 2G, H, J). More importantly, when we set the optimal cut-offs of ISH (ISH score ≥ 3 as high and ISH score < 3 as low), the tRF-Pro-AGG-004-high and/or tRF-Leu-CAG-002-high PC patients showed significantly worse prognosis (Fig. 2I, K). These results suggested that the ISH score of tRF-Pro-AGG-004 and tRF-Leu-CAG-002 could be valuable biomarkers for predicting survival time of patients after surgery.

### Increased tsRNAs cleavaged by angiogenin in pancreatic cancer

**Fig. 3:**
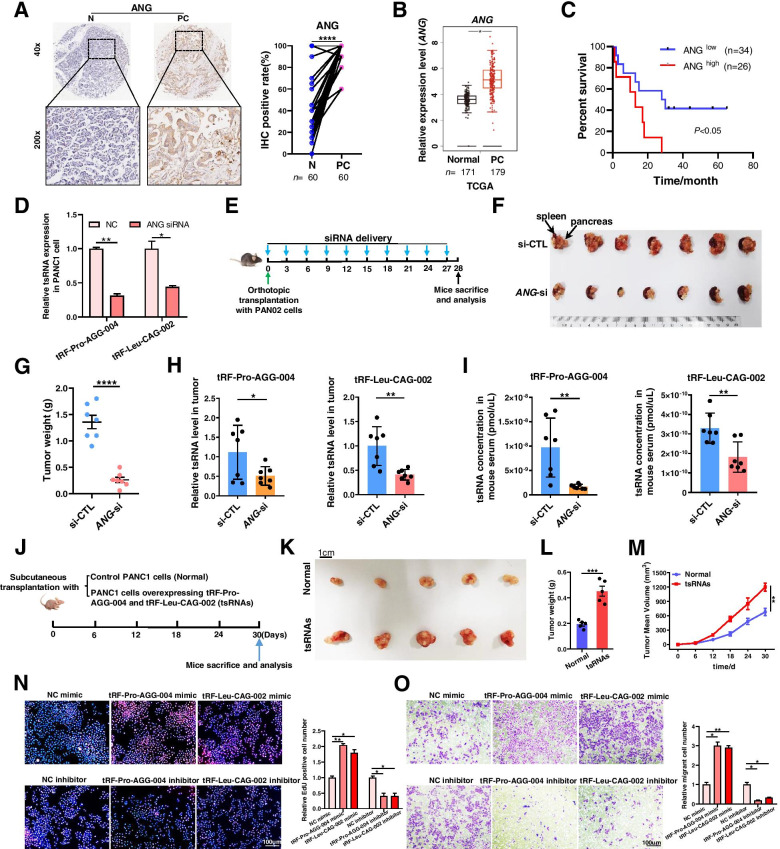
The production mechanisms and biological functions of the two tsRNAs. **A** Representative images (left) and statistics analysis (right) of ANG staining from 60 PC tissues (PC) and 60 matched normal adjacent tissue (N). **B** TCGA dataset analysis of ANG in 179 pancreatic cancerous tissues (PC) and 171 normal pancreatic tissues (Normal). **C** For survival analyses, IHC intensities of ANG protein in PC tissues and paired normal adjacent tissues (*n* = 60) were collected and analyzed based on the ratio of IHC intensities (PC tissues/ paired normal adjacent tissues). The median of the data set was calculated as 1.11. And the median value 1.11 was set as cut-off value, > 1.11 as high expression and ≤ 1.11 as low expression. **D** PANC1 cells were transfected with control siRNA or ANG siRNA. Total RNA was extracted and subjected to qRT-PCR detection of tRF-Pro-AGG-004 and tRF-Leu-CAG-002. **E**-**I** Intravenous injection of ANG siRNA inhibited tumor growth and tsRNAs expression in vivo. **E** Flow chart of the experimental design. The PC orthotopic transplantation model was constructed using PAN02 cells in C57BL/6 J mice. Then the mice were randomly divided into two groups and intravenous delivered cholesterol-modified ANG siRNA (ANG-si) or control siRNA (si-CTL) every 3 days for 4 weeks (7 mice/group). **F** Tumor image at the day 28. **G** Quantitative analysis of tumor weights. **H**, **I** Quantitative analysis of two tsRNAs levels in tumor and serum in mice from two groups. **J**-**O** tRF-Pro-AGG-004 and tRF-Leu-CAG-002 function in pancreatic cancer. **J** Flow chart of the experimental design. PANC1 cells infected with control lentivirus (Normal), tRF-Pro-AGG-004 and tRF-Leu-CAG-002 overexpression lentivirus (tsRNAs) were implanted subcutaneously into nude mice (5 mice/group), and tumor growth was evaluated on day 30 after implantation. **K** Tumor images. **L**, **M** Quantitative analysis of tumor weights and tumor volumes. **N**, **O** Representative images and histogram statistics from EdU and invasion assays of PANC1 cells transfected with tsRNA mimics or inhibitors. *P < 0.05; **P < 0.01; ****P < 0.0001

To further explore mechanisms of the two tsRNAs production, we measured the major enzymes involved in tsRNAs processing, including ANG and Dnmt2 [5] in 60 paired PC tumor and normal tissues by IHC chip. ANG was greatly elevated in 34/60 (56.7%) PC cases, whereas Dnmt2 showed no significant difference between two groups (Fig. 3A, Fig. S7). TCGA analysis also generated similar results (Fig. 3B). These results indicated that the upregulated tRF-Pro-AGG-004 and tRF-Leu-CAG-002 in tumor tissues may be produced due to increased cleavage by ANG. Notably, PC patients with higher ANG showed markedly shorter overall survival time (Fig. 3C). To determine whether ANG is required for the production of tsRNAs in PC, we silenced ANG expression in PANC1 cells (Fig. S8A). Strikingly, transfection with ANG siRNA resulted in the reduction of tRF-Pro-AGG-004 and tRF-Leu-CAG-002 in PC cells and culture medium (Fig. 3D, Fig. S8B). Moreover, depletion of ANG by using in-vivo-optimized RNAi significantly reduced tumor and serum tsRNAs levels in PC orthotopic transplantation tumor model (Fig. 3E-I). These results indicated that upregulated tsRNAs may be produced through increased cleavage by ANG and ANG might be a potential target for PC therapy.

### tRF-Pro-AGG-004 and tRF-Leu-CAG-002 function in pancreatic cancer

Previous studies found that some tsRNAs may be structurally and functionally similar to miRNA, directly binding to target mRNA and resulting in translational repression [6]. Therefore, we used RNAhybrid, GO and KEGG to analyze target genes and associated biological processes. The top 10 high-enrichment GO terms targeted by the two tsRNAs included cell–cell adhesion via plasma-membrane adhesion molecules, Rho protein signal transduction, Hippo signaling pathway, etc. KEGG showed that CAMs were significantly enriched (Fig. S9, Tables S7 and S8). Particularly, Rho GTPases, Hippo pathway, CAMs have been implicated in tumor progression. These bioinformatics interpretations suggested that the two tsRNAs may have regulatory effects on tumorigenesis and progression by affecting various signaling pathways.

Next, we evaluated the functional significance of two tsRNAs in pancreatic cancer by in vivo and in vitro assays. PANC1 cells were infected with tRF-Pro-AGG-004 and tRF-Leu-CAG-002 overexpression lentivirus simultaneously (Fig. S10). Next, we subcutaneously implanted the infected cells into six-week-old nude mice. Tumor growth were evaluated 30 days after cell implantation (Fig. 3J,K). The weights or volumes of xenografted tumors were higher or larger in the tsRNA-overexpressing group than in the control group (Fig. 3L,M). Moreover, EdU assays revealed that the tsRNAs mimics accelerated PANC1 proliferation, whereas the inhibitors delayed cell proliferation (Fig. 3N). Transwell assays showed that tsRNAs overexpression promoted cell invasion, whereas two tsRNAs inhibition inhibited invasion (Fig. 3O). Taken together, these data indicated that the two tsRNAs play a tumor promoter role in pancreatic cancer.

## Conclusions

In summary, we first identified a novel serum two-tsRNAs signature in PC patients. We then demonstrated that serum tRF-Pro-AGG-004 and tRF-Leu-CAG-002 could be used as novel promising biomarkers for PC diagnosis, even in early stage. Furthermore, our results showed that the ISH score of tRF-Pro-AGG-004 and tRF-Leu-CAG-002 in tumor tissues could be valuable biomarkers for predicting survival time of patients after surgery. Our study also revealed the origination, existence forms and potential biological functions of the two novel serum tsRNAs. Randomized clinical trials will be needed to evaluate the possible application of the two-tsRNAs signature in the early diagnosis and prognosis of pancreatic cancer.

## Supplementary Information

**Additional file 1: Fig. S1.** Analysis of small RNAs in serum pools in PC patients and normal healthy controls. **Fig. S2.** Analysis of differentially expressed serum tsRNAs in pancreatic cancer. **Fig. S3.** ROC curves for serum tRF-Pro-AGG-004 and tRF-Leu-CAG-002 in the training cohort. **Fig. S4.** CA19-9 and CEA concentrations in serum samples from patients with pancreatic cancer versus healthy controls. **Fig. S5.** tRF-Pro-AGG-004 and tRF-Leu-CAG-002 expression in pancreas tissues from PC mice. **Fig. S6.** Increased tRF-Pro-AGG-004 and tRF-Leu-CAG-002 expression in PC tissues. **Fig. S7.** Dnmt2 expression in human pancreatic cancer tissues. **Fig. S8.** ANG knockdown in PC cells decreased tRF-Pro-AGG-004 and tRF-Leu-CAG-002 expression. **Fig. S9.** Top-10 GO annotation and KEGG terms of the biological pathways of tRF-Pro-AGG-004 and tRF-Leu-CAG-002 target genes. **Fig. S10.** The overexpression efficiency of tsRNAs lentivirus on tsRNA levels.

**Additional file 2.** Materials and Methods.

**Additional file 3: Table S1.** The 26 significant differentially expressed tsRNAs in PC serum compared with healthy controls. **Table S2.** Clinical significance of serum tRF-Pro-AGG-004 and tRF-Leu-CAG-002 expression in serum from 204 patients with pancreatic cancer. **Table S3.** The clinical features of samples with other types of diseases. **Table S4.** Clinical features of 20 PC patients. **Table S5.** Clinicopathologic characteristics of tRF-Pro-AGG-004 and tRF-Leu-CAG-002 expression in cohort 1 (60 PC patients). **Table S6.** Clinicopathologic characteristics of tRF-Pro-AGG-004 and tRF-Leu-CAG-002 expression in cohort 2 (75 PC patients). **Table S7.** tRF-Pro-AGG-004 pathway enrichment analysis. **Table S8.** tRF-Leu-CAG-002 pathway enrichment analysis.

## Data Availability

All data generated or analyzed during this study are included in this published article [and its supplementary information]. The small RNA sequence data have been uploaded to NCBI Sequence Read Archive (SRA) (Accession Number: PRJNA744375).

## Abbreviations

PC: Pancreatic cancer
tsRNA: TRNA-derived small RNAs
CPM: Counts per million
AUC: Area under the receiver operating characteristic curve
TNM: Tumor-node-metastasis
ANG: Angiogenin
Dnmt2: DNA methyltransferase 2
IHC: Immunohistochemical
ISH: In situ hybridization
CAMs: Cell adhesion molecules
