# Supplementary material for: A novel class of tsRNA signatures as biomarkers for diagnosis and prognosis of pancreatic cancer
Source: Mol Cancer. 2021 Jul 17;20:95. doi: 10.1186/s12943-021-01389-5 (PMC8285832; doi:10.1186/s12943-021-01389-5)
Supplement: Supplementary file 1 — Additional file 1: Fig. S1. Analysis of small RNAs in serum pools in PC patients and normal healthy controls. Fig. S2. Analysis of differentially expressed serum tsRNAs in pancreatic cancer. Fig. S3. ROC curves for serum tRF-Pro-AGG-004 and tRF-Leu-CAG-002 in the training cohort. Fig. S4. CA19-9 and CEA concentrations in serum samples from patients with pancreatic cancer versus healthy controls. Fig. S5. tRF-Pro-AGG-004 and tRF-Leu-CAG-002 expression in pancreas tissues from PC mice. Fig. S6. Increased tRF-Pro-AGG-004 and tRF-Leu-CAG-002 expression in PC tissues. Fig. S7. Dnmt2 expression in human pancreatic cancer tissues. Fig. S8. ANG knockdown in PC cells decreased tRF-Pro-AGG-004 and tRF-Leu-CAG-002 expression. Fig. S9. Top-10 GO annotation and KEGG terms of the biological pathways of tRF-Pro-AGG-004 and tRF-Leu-CAG-002 target genes. Fig. S10. The overexpression efficiency of tsRNAs lentivirus on tsRNA levels. [file 12943_2021_1389_MOESM1_ESM.pdf]

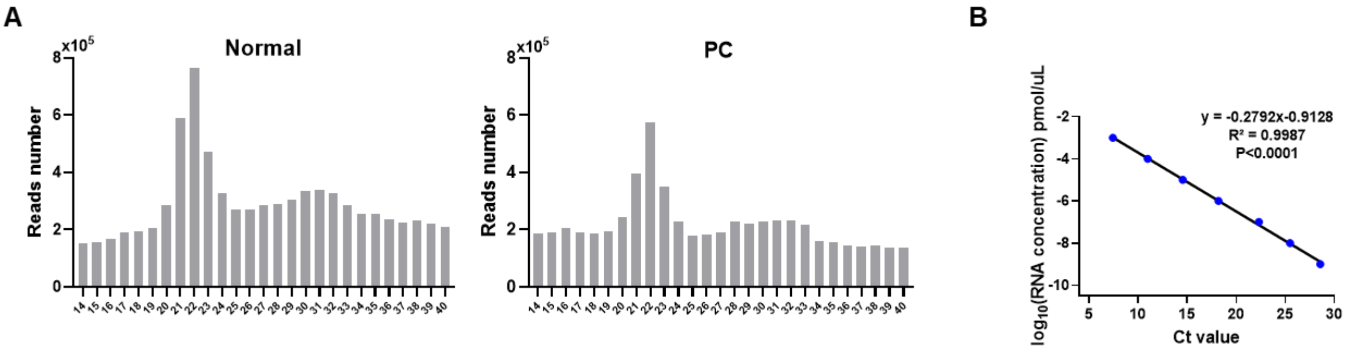

Fig. S1 Analysis of small RNAs in serum pools in PC patients and normal healthy controls. A Length distribution of small RNAs in serum pools from 30 PC patients and 30 normal controls. **B** The representative standard curve of TaqMan-custom synthesised probe-based RT-qPCR for tRF-Pro-AGG-004. RNA concentration represents the synthetic tRF-Pro-AGG-004 concentration before reverse transcription.

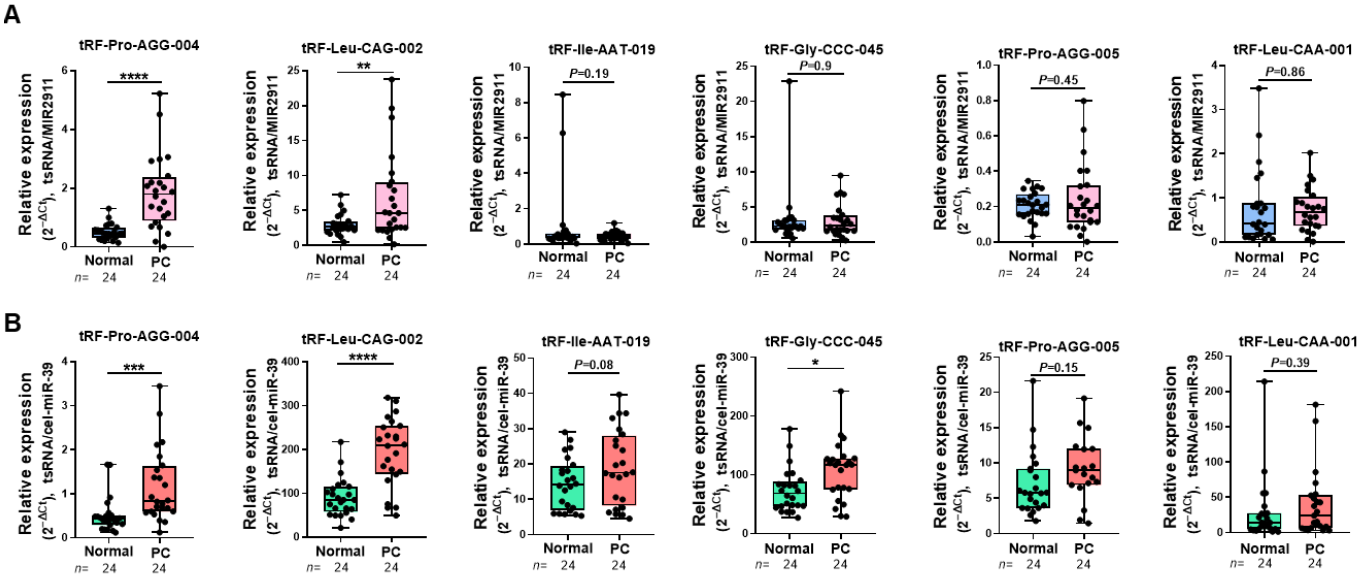

Fig. S2 Analysis of differentially expressed serum tsRNAs in pancreatic cancer. Expression of six serum tsRNAs in PC patients in training set. Serum samples from 24 PC patients and 24 controls were collected and subjected to qRT-PCR relative quantification. **A, B** Plant miRNA MIR2911 (A) and *C. elegans* miRNA cel-miR-39 (B) serve as exogenous reference genes. *$P < 0.05$; **$P < 0.01$; ***$P < 0.001$, ****$P < 0.0001$.

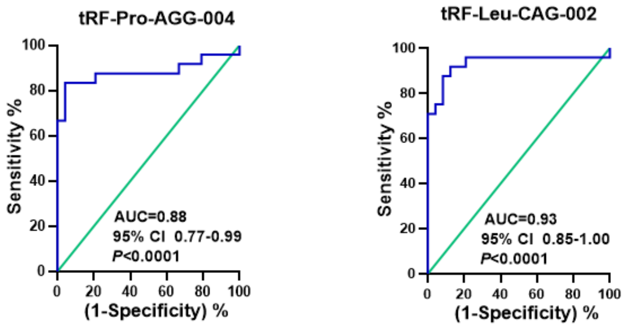

Fig. S3 ROC curves for serum tRF-Pro-AGG-004 and tRF-Leu-CAG-002 in the training cohort.

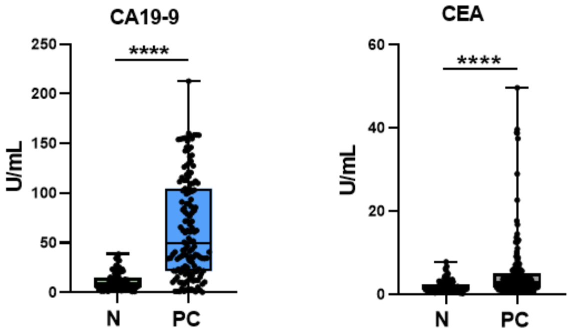

Fig. S4 CA19-9 and CEA concentrations in serum samples from patients with pancreatic cancer versus healthy controls. ELISA assay shows the concentrations of CA19-9 and CEA in serum samples of PC patients and healthy controls. ****P < 0.0001.

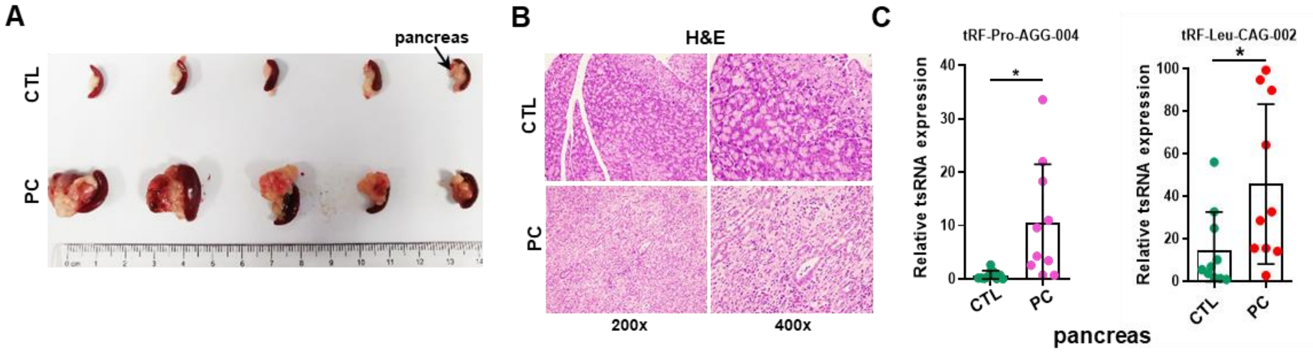

Fig.S5 tRF-Pro-AGG-004 and tRF-Leu-CAG-002 expression in pancreas tissues from PC mice. A Representative images of spleen and pancreas from normal mice and PC orthotopic transplantation tumor mice on day 35 after implantation. **B** HE staining of tumor and normal pancreatic tissues. **C** tRF-Pro-AGG-004 and tRF-Leu-CAG-002 expression status in pancreas tissues from two groups (*n*=10). *$P < 0.05$.

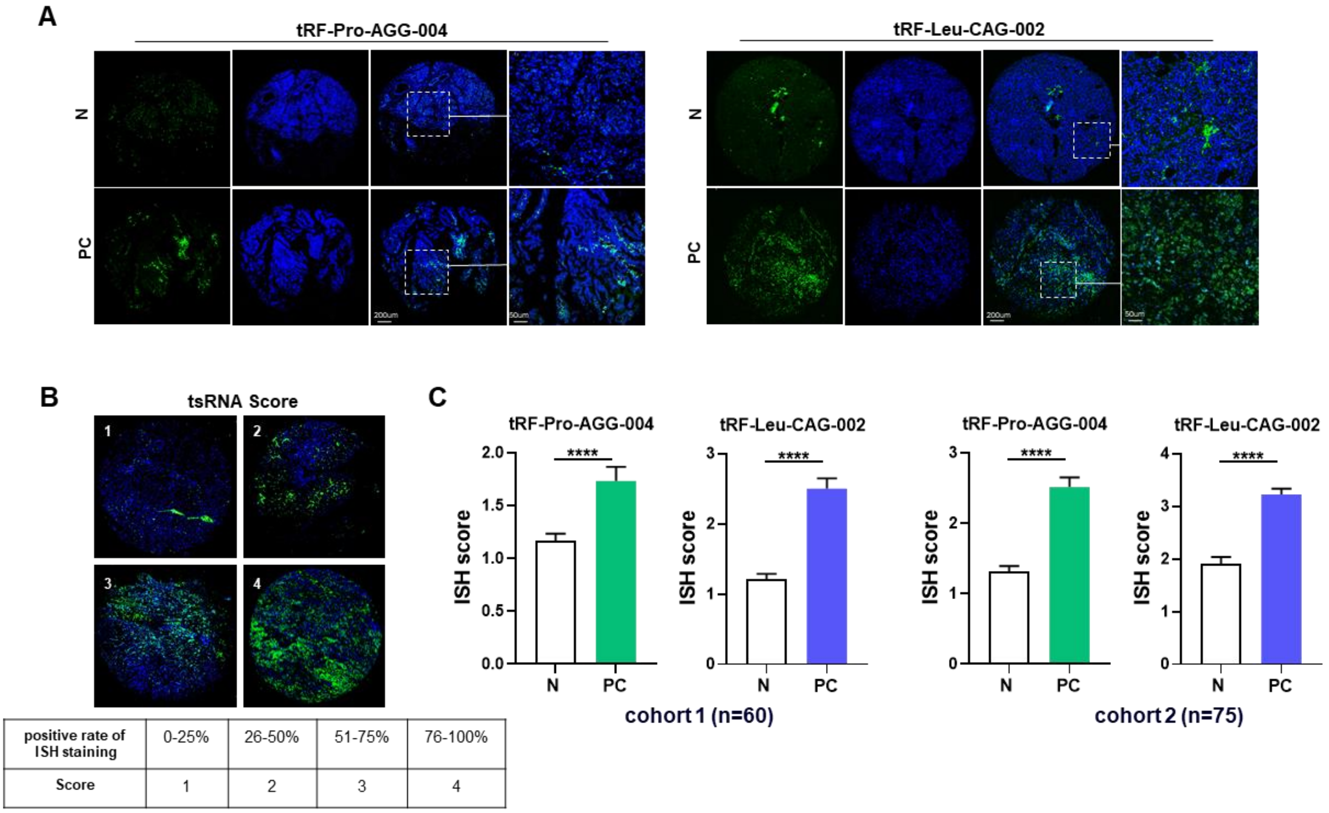

positive rate of ISH staining	0-25%	26-50%	51-75%	76-100%
Score	1	2	3	4

Fig.S6 Increased tRF-Pro-AGG-004 and tRF-Leu-CAG-002 expression in PC tissues. A Representative results for *in situ* hybridization (ISH) staining of tRF-Pro-AGG-004 or tRF-Leu-CAG-002 in pancreatic cancer (PC) and corresponding normal tissues (N). **B** According to positive rate of ISH staining, each sample was scored as 1(0-25%), 2(26-50%), 3(51-75%), 4(76-100%). **C** ISH scores of tRF-Pro-AGG-004 or tRF-Leu-CAG-002 in pancreatic cancer (PC) and corresponding normal tissues (N) from 2 cohorts. ****$P < 0.0001$.

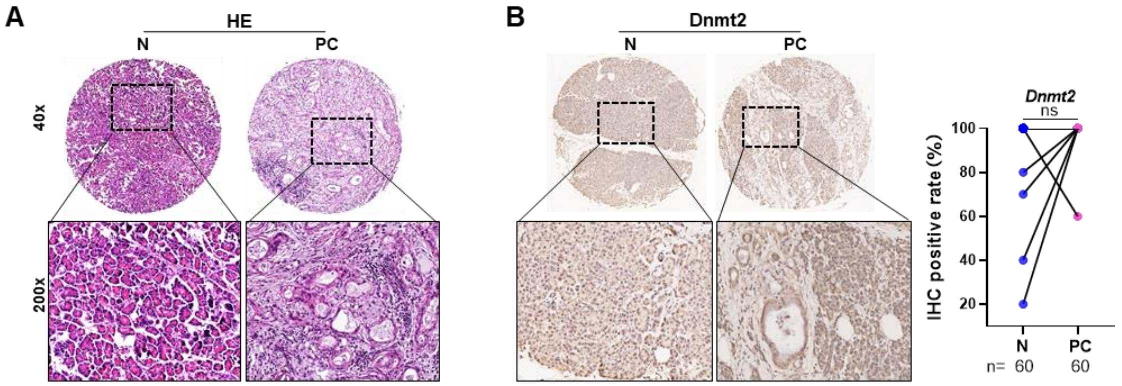

Fig.S7 Dnmt2 expression in human pancreatic cancer tissues. A Representative images of HE staining. **B** Representative image and statistical analysis of Dnmt2 staining from 60 PC tissues (PC) and 60 matched normal adjacent tissue (N). ns, P > 0.05.

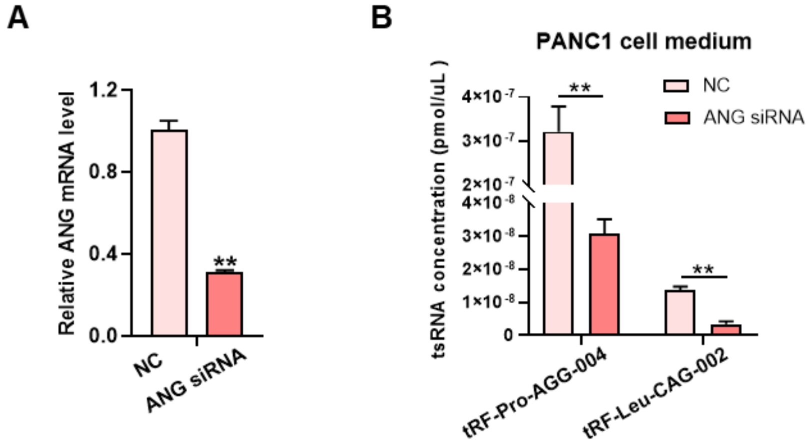

Fig. S8 ANG knockdown in PC cells decreased tRF-Pro-AGG-004 and tRF-Leu-CAG-002 expression. A Knockdown of ANG mRNA by siRNA. siRNA designed to target the ANG gene were effective to decrease ANG mRNA levels. **B** tRF-Pro-AGG-004 and tRF-Leu-CAG-002 levels in culture medium after ANG siRNA transfection. **P < 0.01.

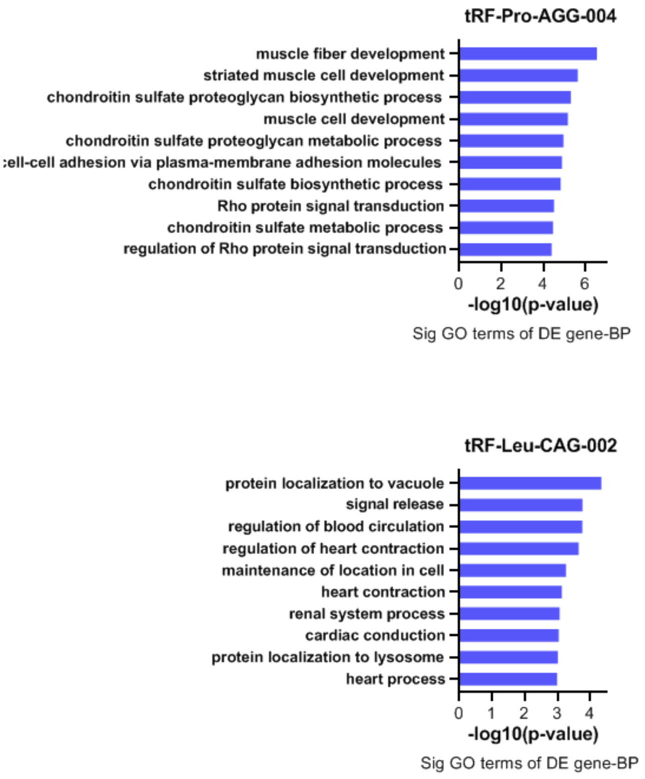

Fig. S9 Top-10 GO annotation and KEGG terms of the biological pathways of tRF-Pro-AGG-004 and tRF-Leu-CAG-002 target genes.

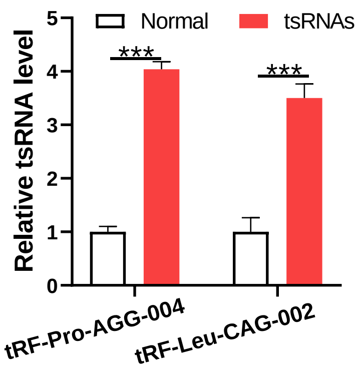

Fig. S10 The overexpression efficiency of tsRNAs lentivirus on tsRNA levels. QRT-PCR analysis of tRF-Pro-AGG-004 and tRF-Leu-CAG-002 expression levels in PANC1 cells after infection with lentiviral expression vectors of tRF-Pro-AGG-004 and tRF-Leu-CAG-002, simultaneously. U6 served as the internal reference. ***P < 0.001.
