# Supplementary material for: A novel class of tsRNA signatures as biomarkers for diagnosis and prognosis of pancreatic cancer
Source: Mol Cancer. 2021 Jul 17;20:95. doi: 10.1186/s12943-021-01389-5 (PMC8285832; doi:10.1186/s12943-021-01389-5)
Supplement: Supplementary file 2 — Additional file 2. Materials and Methods. [file 12943_2021_1389_MOESM2_ESM.docx]

**Materials and Methods**

**Clinical samples**

All the serum samples were collected before any therapeutic procedures in The First People’s Hospital of Lianyungang (100 PC patients, 80 healthy controls) and The Affiliated Drum Tower Hospital of Nanjing University Medical School (104 PC patients, 74 healthy controls) from 2016.06 to 2020.01. All serum samples were collected using standard procedures and stored at -80°C until analysis. The patients had performed a biopsy procedure or surgical resection for pathological diagnosis and they have no previous pancreatic cancer and other cancers. None of these patients received preoperative cancer treatment (chemotherapy or radiotherapy). Tumors were classified based on the Seventh Edition of the AJCC tumor-node-metastasis staging system. The healthy controls were recruited at the Healthy Physical Examination Center of two hospitals. The health examination checkup included routine blood tests, physical, radiological, tumor marker tests and endoscopic examinations. Subjects with no abnormal physiological and pathologic factors were enrolled as cancer-free controls. The clinical pathological characteristics of the patients are described in Table S2. The clinical pathological characteristics of 239 patients (31 PC, 48 HCC, 60 BRC, 53 NSCLC, 23 hepatocirrhosis and 24 hepatitis) obtained from The Affiliated Drum Tower Hospital of Nanjing University Medical School are described in Table S3. Tissue microarray (TMA) chips and In situ hybridization (ISH) chips containing cohort 1 (n=60, underwent surgery in 2009.05-2013.06) and cohort 2 (n=75, underwent surgery in 2004.09-2008.12) were obtained from Shanghai Zhangjiang Biobank (Shanghai, China) and clinical pathological characteristics of the patients are described in Table S4, 5. Fresh human PC surgical specimens and paired serum were obtained from 20 patients at The First People’s Hospital of Lianyungang (Table S6). All experiments were approved by the Medical Ethics Committee of The First People’s Hospital of Lianyungang and The Affiliated Drum Tower Hospital of Nanjing University Medical School.

**Quantitative RT-PCR for tsRNAs**

Total RNA was extracted from 100 μL serum and dissolved in 20 μL DEPC water as we previously described [[1](#_ENREF_1)]. TaqMan probe-based RT-qPCR assay was carried out using a commercial kit (Applied Biosystems, Foster City, CA, USA). Briefly, 2 μL of total RNA was reverse transcribed to cDNA using AMV reverse transcriptase (TaKaRa, Dalian, China) and the stem-loop RT primer (synthesised by Applied Biosystems, USA). The mix was incubated at 16 °C for 15 min, 42 °C for 60 min, and 85 °C for 5 min. Subsequently, real-time quantification was performed using qRT-PCR was performed on a Roche LightCycler 480 II RT-PCR System (Roche, Germany) using TaqMan-custom synthesised tsRNA probes (Applied Biosystems) following the instructions. The PCR procedure for tsRNA was as follows, 95 °C, 10 min, followed by 45 cycles at 95 °C for 15 s, 60 °C for 1 min. The threshold cycle (C_t_) values were determined using the fixed threshold settings.

For the absolute quantitative analysis of tsRNAs, a series of synthetic tsRNA oligonucleotides (dissolved in DEPC water) of known concentrations (from 10 fmol/L to 10 nmol/L) were also reverse-transcribed and amplified to generate a standard curve. The absolute amount of tsRNA was then calculated by referring to the standard curve. For the relative quantitative analysis, the relative levels of tsRNAs were normalized to an exogenous reference gene MIR-2911(a plant-derived miRNA) and calculated using the 2^−△Ct^ method. As there is no current consensus on housekeeping tsRNAs for qRT-PCR analysis of serum tsRNAs, the expression levels of tsRNAs were directly normalized to serum volume in this study.

**Small RNA sequencing**

*RNA Sample preparation*

Before the sequencing experiment, we check the integrity and quantity of each RNA sample using agarose gel electrophoresis and NanodropTM instrument. The serum samples were pooled from 30 PC patients or 30 healthy controls. The serum was added with TRIzol reagent and homogenized, followed by RNA extraction.

*Pretreatment* *of tsRNAs*

tsRNAs are heavily decorated by RNA modifications that interfere with small RNA-seq library construction. We do the following treatments before library preparation for total RNA samples: 3’-aminoacyl (charged) deacylation to 3’-OH for 3’adaptor ligation, 3’-cP (2’,3’-cyclic phosphate) removal to 3’-OH for 3’adaptor ligation, 5’-OH (hydroxyl group) phosphorylation to 5’-P for 5’-adaptor ligation, m1A and m3C demethylation for efficient reverse transcription.

*sRNA library construction and sequencing*

All sRNA library construction and deep sequencing were performed by Aksomics (Shanghai, China). Sequencing libraries are size-selected for the RNA biotypes to be sequenced using an automated gel cutter. The libraries are qualified and absolutely quantified using Agilent BioAnalyzer 2100. sRNA libraries were constructed according to the Agilent BioAnalyzer 2100. For standard small RNA sequencing on Illumina NextSeq instrument, the sequencing type is 50bp single read.

*Data processing and analysis*

tRNA sequences of cytoplasmic were downloaded from GtRNAdb [[2](#_ENREF_2)] . tRNA sequences of mitochondrial were predicted with tRANscan-SE [[3](#_ENREF_3)] software. To generate the mature tRNA libraries, we removed the predicted intronic sequences (if present) and added an additional 3’-terminal “CCA” to each tRNA. To generate the precursor tRNA libraries, we included 40 nucleotides of flanking genomic sequence on either side of the original tRNA sequence [[4](#_ENREF_4)]. Besides, Precursor and mature miRNA sequences, tRNA sequences were obtained from miRBase v21 [[5](#_ENREF_5)]. For normalization, the total sequencing frequency of each type of sRNA in each sample was normalized to 1,000,000. Differential analysis was performed using Student’s t test. Significance was set at uncorrected *P* < 0.05 for broad pattern identification. A fold-change threshold was set at > 2.

***In situ* hybridisation**

*In situ* hybridization (ISH) was conducted on slides containing pancreatic cancer tissues (PC) and matched normal tissues (N) from 60 patients using an locked nucleic acid (LNA) oligonucleotide probes against tRF-Pro-AGG-004 and tRF-Leu-CAG-002 with 5′-DIG-labelled and 3′-DIG-labelled oligonucleotide (Exiqon, Woburn, Massachusetts, USA).The results were quantified as previously described [[6](#_ENREF_6), [7](#_ENREF_7)]. Finally, each sample was scored as 1-4 [1(0-25%), 2(26-50%), 3(51-75%), 4(76-100%)] according to positive rate of ISH staining. Then we set the optimal cut-offs of ISH, ISH score≥3 as high and ISH score＜3 as low. For survival analyses, the follow-up data of PC patients were collected and analyzed based on ISH scores of tsRNAs.

**Tissue microarray analysis**

Tissue microarrays (TMA) including 60 pairs of PC samples (PC) and paired normal adjacent tissues (N) were performed. Immunohistochemical staining intensities of ANG and DNMT2 were collected by ImageJ software. Based on ANG/Dnmt2 positive rate (ANG/ Dnmt2-positive cells/all cells), we obtained the values with a scale of 0%~100%. For survival analyses, IHC intensities of ANG protein in PC tissues and paired normal adjacent tissues (n=60) were collected and analyzed based on the ratio of IHC intensities (PC tissues/ paired normal adjacent tissues). We calculated the median of the data set and get the median value as 1.11. Then we set the median value 1.11 as cut-off value, >1.11 as high expression and ≤ 1.11 as low expression. In this data set, 26 values >1.11, 27 values <1.11 and 7 values=1.11, so we set n=26 for high and n=34 for low.

**Cell lines**

Human pancreatic cancer cell lines PANC-1 and mouse pancreatic cancer cell line Pan02 were obtained from ATCC (Maryland, USA) for *in vitro* analysis and maintained in DMEM supplemented with 10% FBS (Gibco) at 37°C with 5% CO2.

**CA19-9 and CEA Elisa**

Serum CA19-9 and CEA levels in patients with pancreatic cancer and healthy controls were assessed using the cancer antigen CA19-9 human ELISA kit (Abcam, ab108642) and the CEA human ELISA kit (Biovision, K4805-100), according to the manufacturer’s directions.

**Cell proliferation and invasion assay**

Cell proliferation was determined by EdU Cell Proliferation Assay Kit (RiboBio, Guangzhou, China) and the polycarbonate membrane inserts (Corning, NY, USA) coated with matrigel were used to analyze the invasive ability of cells as we previously described [[8](#_ENREF_8), [9](#_ENREF_9)].

**Animal experiments**

Six-week-male nude mice and C57BL/6J mice were purchased from the Model Animal Research Center, Nanjing University (Nanjing, China). For orthotopic injection of pancreatic cancer cells, mice were anesthetized by pentobarbitone and the abdomen was sterilized. Then 5-10 mm laparotomy was performed over the left upper quadrant of the abdomen to expose the peritoneal cavity. The pancreas was exteriorized onto a sterile field, and sterile PBS or pancreatic tumor cells PAN02 (1×10^6^ cells/100 μL) were injected into the pancreas (10 mice/group). Successful injection was verified by the formation of a liquid bleb at the injection site with minimal fluid leakage. The pancreas was then gently placed back into the peritoneal cavity. Mice orthotopically injected with PAN02 were sacrificed after 5 weeks, pancreas tissues were resected, fixed in 4% paraformaldehyde, embedded in paraffin and sectioned at 5 mm for H&E. Mice serum and tumor were isolated for tsRNAs expression analysis. For xenograft subcutaneous implantations, PANC1 cells (2× 10^6^ cells/ 100 μL) were injected into oxter of nude mice (5 mice/group). Tumor growth rate was monitored every 6 days. Mice were sacrificed after 30 days, and tumours were collected, weighed and analyzed. All experiments were approved by the National Institutes of Health guide for the care and use of mice and were approved by IACUC, Nanjing University (Nanjing, China).

**Data statistics**

All statistical tests were performed using GraphPad Prism software 8 (San Diego, CA). Data are presented as means ± SEMs. Differences are considered statistically significant at *P* < 0.05. Normality and equal variances between group samples were assessed using Shapiro-Wilk test and Brown–Forsythe tests, respectively. When normality and equal variance was achieved between sample groups, one-way ANOVA (followed by Bonferroni’s multiple comparisons test), two-way ANOVA (followed by Bonferroni’s multiple comparisons test) or t-test were used.

**References**

1. Chen X, Ba Y, Ma L, Cai X, Yin Y, Wang K, Guo J, Zhang Y, Chen J, Guo X, et al: **Characterization of microRNAs in serum: a novel class of biomarkers for diagnosis of cancer and other diseases.** *Cell Research* 2008, **18:**997-1006.

2. Chan PP, Lowe TM: **GtRNAdb 2.0: an expanded database of transfer RNA genes identified in complete and draft genomes.** *Nucleic Acids Research* 2016, **44:**D184-D189.

3. Lowe TM, Chan PP: **tRNAscan-SE On-line: integrating search and context for analysis of transfer RNA genes.** *Nucleic Acids Research* 2016, **44:**W54-W57.

4. Selitsky SR, Sethupathy P: **tDRmapper: challenges and solutions to mapping, naming, and quantifying tRNA-derived RNAs from human small RNA-sequencing data.** *Bmc Bioinformatics* 2015, **16**.

5. Kozomara A, Griffiths-Jones S: **miRBase: annotating high confidence microRNAs using deep sequencing data.** *Nucleic Acids Research* 2014, **42:**D68-D73.

6. Li A, Yu J, Kim H, Wolfgang CL, Canto MI, Hruban RH, Goggins M: **MicroRNA Array Analysis Finds Elevated Serum miR-1290 Accurately Distinguishes Patients with Low-Stage Pancreatic Cancer from Healthy and Disease Controls.** *Clinical Cancer Research* 2013, **19:**3600-3610.

7. Lu J, Tsourkas A: **Imaging individual microRNAs in single mammalian cells in situ.** *Nucleic Acids Research* 2009, **37:**10.

8. Liu Y, Liu R, Yang F, Cheng R, Chen X, Cui S, Gu Y, Sun W, You C, Liu Z, et al: **miR-19a promotes colorectal cancer proliferation and migration by targeting TIA1.** *Molecular Cancer* 2017, **16**.

9. Liu Y, Chen X, Cheng R, Yang F, Yu M, Wang C, Cui S, Hong Y, Liang H, Liu M, et al: **The Jun/miR-22/HuR regulatory axis contributes to tumourigenesis in colorectal cancer.** *Molecular Cancer* 2018, **17**.
