# Supplementary material for: A novel class of tsRNA signatures as biomarkers for diagnosis and prognosis of pancreatic cancer
Source: Mol Cancer. 2021 Jul 17;20:95. doi: 10.1186/s12943-021-01389-5 (PMC8285832; doi:10.1186/s12943-021-01389-5)
Supplement: Supplementary file 3 — Additional file 3: Table S1. The 26 significant differentially expressed tsRNAs in PC serum compared with healthy controls. Table S2. Clinical significance of serum tRF-Pro-AGG-004 and tRF-Leu-CAG-002 expression in serum from 204 patients with pancreatic cancer. Table S3. The clinical features of samples with other types of diseases. Table S4. Clinical features of 20 PC patients. Table S5. Clinicopathologic characteristics of tRF-Pro-AGG-004 and tRF-Leu-CAG-002 expression in cohort 1 (60 PC patients). Table S6. Clinicopathologic characteristics of tRF-Pro-AGG-004 and tRF-Leu-CAG-002 expression in cohort 2 (75 PC patients). Table S7. tRF-Pro-AGG-004 pathway enrichment analysis. Table S8. tRF-Leu-CAG-002 pathway enrichment analysis. [file 12943_2021_1389_MOESM3_ESM.pdf]

Table S1. The 26 significant differentially expressed tsRNAs in PC serum compared with healthy controls.

	tRF_ID	Type	Length	CPM Normal	CPM PC	Fold Change (PC/Normal)
1	tRF-Pro-AGG-004	tRF-5c	30	16.27127	1126.97	69.26132
2	tRF-Gln-CTG-034	tRF-2	14	24.40691	791.2768	32.42019
3	tRF-Leu-CAG-002	tRF-3a	18	16.27127	341.6877	20.99944
4	tRF-Ile-AAT-019	tRF-3a	18	16.27127	257.7644	15.84168
5	tRF-Gly-CCC-045	tRF-3a	18	12.20346	191.8247	15.71888
6	tRF-Pro-AGG-005	tRF-5c	31	48.81382	701.359	14.36804
7	tRF-Thr-CGT-009	tRF-5a	15	28.47473	365.6658	12.84176
8	tRF-Pro-AGG-002	tRF-5c	28	16.27127	179.8356	11.05234
9	tRF-Leu-CAA-001	tRF-3a	18	40.67819	443.5946	10.90497
10	tRF-Ser-AGA-005	tRF-5b	23	248.1369	23.97808	0.096632
11	tRF-Val-AAC-006	tRF-5a	16	374.2393	35.96713	0.096107
12	tRF-Gly-GCC-032	tRF-5a	16	138.3058	11.98904	0.086685
13	tRF-Pro-AGG-011	tRF-3b	19	414.9175	29.97261	0.072238
14	tRF-Ser-AGA-004	tRF-5a	16	508.4773	35.96713	0.070735
15	tRF-Gly-TCC-016	tRF-5c	32	187.1197	11.98904	0.064072
16	tRF-Ile-TAT-003	tRF-3a	18	191.1875	11.98904	0.062708
17	tiRNA-Lys-TTT-001	tiRNA-5	29	203.3909	11.98904	0.058946
18	tRF-Gly-TCC-055	tRF-1	14	215.5944	11.98904	0.055609
19	tRF-Ala-AGC-057	tRF-3b	22	227.7978	11.98904	0.05263
20	tRF-Glu-TTC-025	tRF-5c	32	227.7978	11.98904	0.05263
21	tRF-Val-CAC-005	tRF-5b	23	244.0691	11.98904	0.049122
22	tRF-Ser-GCT-003	tRF-1	17	370.1715	17.98356	0.048582
23	tRF-Leu-CAG-019	tRF-3b	22	252.2048	11.98904	0.047537
24	tRF-Ser-GCT-005	tRF-5a	14	3522.731	125.8849	0.035735
25	tiRNA-Val-CAC-001	tiRNA-5	34	1073.904	11.98904	0.011164
26	tRF-Ser-GCT-024	tRF-5a	15	27087.6	275.748	0.01018

Table S2. Clinical significance of serum tRF-Pro-AGG-004 and tRF-Leu-CAG-002 expression in serum from 204 patients with pancreatic cancer.

		Serum tRF-Pro-AGG-004		Serum tRF-Leu-CAG-002	
	**n**	**Mean±SD**	**pValue**	**Mean±SD**	**p Value**
**Sex**			0.1039		0.5056
Male	127	5.80E-08±7.48E-08		2.93E-07±1.28E-06	
Female	77	3.94E-08±4.90E-08		1.77E-07± 2.20E-07	
**Age(years)**			0.1947		0.4576
<Median(62)	86	4.99E-08±6.51E-08		1.76E-07±2.21E-07	
≥Median(62)	118	6.91E-08±1.02E-07		3.03E-07±1.33E-06	
**Metastasis**			5.61E-06		0.0069
Negative	177	5.52E-08±7.98E-08		2.56E-07±1.07E-06	
Positive	27	1.75E-07 ± 1.32E-07		9.04E-07±2.27E-06	

Table S3. The clinical features of samples with other types of diseases.

Case No.	Age	Gender	Cancer subtype	Case No.	Age	Gender	Cancer subtype
1	63	F	pancreaticductal adenocarcinoma	43	42	M	hepatocellular carcinoma
2	51	M	pancreatic ductal adenocarcinoma	44	30	M	hepatocellular carcinoma
3	65	M	pancreatic ductal adenocarcinoma	45	39	M	hepatocellular carcinoma
4	84	M	pancreatic ductal adenocarcinoma	46	52	F	hepatocellular carcinoma
5	70	M	pancreatic ductal adenocarcinoma	47	47	M	hepatocellular carcinoma
6	49	M	pancreatic ductal adenocarcinoma	48	44	M	hepatocellular carcinoma
7	52	M	pancreatic ductal adenocarcinoma	49	47	M	hepatocellular carcinoma
8	70	F	pancreatic ductal adenocarcinoma	50	54	M	hepatocellular carcinoma
9	54	M	pancreatic ductal adenocarcinoma	51	74	M	hepatocellular carcinoma
10	63	M	pancreatic ductal adenocarcinoma	52	55	M	hepatocellular carcinoma
11	65	M	pancreatic ductal adenocarcinoma	53	78	M	hepatocellular carcinoma
12	58	M	adenosquamous carcinoma	54	53	F	hepatocellular carcinoma
13	57	F	adenosquamous carcinoma	55	29	M	hepatocellular carcinoma
14	71	M	pancreatic ductal adenocarcinoma	56	65	M	hepatocellular carcinoma
15	56	F	pancreatic ductal adenocarcinoma	57	55	M	hepatocellular carcinoma
16	52	M	pancreatic ductal adenocarcinoma	58	46	M	hepatocellular carcinoma
17	57	M	pancreatic ductal adenocarcinoma	59	53	M	hepatocellular carcinoma
18	51	M	pancreatic ductal adenocarcinoma	60	51	F	hepatocellular carcinoma
19	62	F	pancreatic ductal adenocarcinoma	61	61	F	hepatocellular carcinoma
20	55	F	pancreatic ductal adenocarcinoma	62	60	F	hepatocellular carcinoma
21	46	F	pancreatic ductal adenocarcinoma	63	58	F	hepatocellular carcinoma
22	69	M	pancreatic ductal adenocarcinoma	64	72	M	hepatocellular carcinoma
23	44	M	pancreatic ductal adenocarcinoma	65	53	M	hepatocellular carcinoma
24	64	M	pancreatic ductal adenocarcinoma	66	67	F	hepatocellular carcinoma
25	46	M	pancreatic ductal adenocarcinoma	67	63	F	hepatocellular carcinoma
26	52	F	pancreatic ductal adenocarcinoma	68	53	M	hepatocellular carcinoma
27	72	M	pancreatic ductal adenocarcinoma	69	55	M	hepatocellular carcinoma
28	71	M	pancreatic ductal adenocarcinoma	70	52	M	hepatocellular carcinoma
29	64	F	pancreatic ductal adenocarcinoma	71	62	F	hepatocellular carcinoma
30	62	M	adenosquamous carcinoma	72	38	M	hepatocellular carcinoma
31	77	F	pancreatic ductal adenocarcinoma	73	86	F	hepatocellular carcinoma
32	61	M	hepatocellular carcinoma	74	49	M	hepatocellular carcinoma
33	49	M	hepatocellular carcinoma	75	67	M	hepatocellular carcinoma
34	69	M	hepatocellular carcinoma	76	42	M	hepatocellular carcinoma
35	64	M	hepatocellular carcinoma	77	30	M	hepatocellular carcinoma
36	67	F	hepatocellular carcinoma	78	39	M	hepatocellular carcinoma
37	60	F	hepatocellular carcinoma	79	52	F	hepatocellular carcinoma
38	52	M	hepatocellular carcinoma	80	67	F	breast carcinoma
39	55	M	hepatocellular carcinoma	81	58	F	breast carcinoma
40	45	M	hepatocellular carcinoma	82	56	F	breast carcinoma
41	49	M	hepatocellular carcinoma	83	38	F	breast carcinoma
42	67	M	hepatocellular carcinoma	84	58	F	breast carcinoma

85	72	F	breast carcinoma	129	60	F	breast carcinoma
86	83	F	breast carcinoma	130	65	F	breast carcinoma
87	64	F	breast carcinoma	131	48	F	breast carcinoma
88	68	F	breast carcinoma	132	55	F	breast carcinoma
89	46	F	breast carcinoma	133	39	F	breast carcinoma
90	39	F	breast carcinoma	134	65	F	breast carcinoma
91	45	F	breast carcinoma	135	67	F	breast carcinoma
92	54	F	breast carcinoma	136	47	F	breast carcinoma
93	47	F	breast carcinoma	137	33	F	breast carcinoma
94	51	F	breast carcinoma	138	60	F	breast carcinoma
95	39	F	breast carcinoma	139	36	F	breast carcinoma
96	49	F	breast carcinoma	140	34	F	non-small-cell lung carcinoma
97	46	F	breast carcinoma	141	70	M	non-small-cell lung carcinoma
98	39	F	breast carcinoma	142	36	F	non-small-cell lung carcinoma
99	42	F	breast carcinoma	143	50	F	non-small-cell lung carcinoma
100	64	F	breast carcinoma	144	71	M	non-small-cell lung carcinoma
101	34	F	breast carcinoma	145	66	M	non-small-cell lung carcinoma
102	35	F	breast carcinoma	146	76	M	non-small-cell lung carcinoma
103	48	F	breast carcinoma	147	53	M	non-small-cell lung carcinoma
104	53	F	breast carcinoma	148	52	M	non-small-cell lung carcinoma
105	65	F	breast carcinoma	149	55	M	non-small-cell lung carcinoma
106	36	F	breast carcinoma	150	68	F	non-small-cell lung carcinoma
107	59	F	breast carcinoma	151	65	F	non-small-cell lung carcinoma
108	51	F	breast carcinoma	152	72	F	non-small-cell lung carcinoma
109	51	F	breast carcinoma	153	26	F	non-small-cell lung carcinoma
110	74	F	breast carcinoma	154	59	M	non-small-cell lung carcinoma
111	72	F	breast carcinoma	155	65	F	non-small-cell lung carcinoma
112	83	F	breast carcinoma	156	60	F	non-small-cell lung carcinoma
113	64	F	breast carcinoma	157	70	M	non-small-cell lung carcinoma
114	68	F	breast carcinoma	158	68	M	non-small-cell lung carcinoma
115	46	F	breast carcinoma	159	69	F	non-small-cell lung carcinoma
116	39	F	breast carcinoma	160	79	M	non-small-cell lung carcinoma
117	45	F	breast carcinoma	161	79	M	non-small-cell lung carcinoma
118	54	F	breast carcinoma	162	45	M	non-small-cell lung carcinoma
119	47	F	breast carcinoma	163	55	M	non-small-cell lung carcinoma
120	51	F	breast carcinoma	164	68	M	non-small-cell lung carcinoma
121	39	F	breast carcinoma	165	68	M	non-small-cell lung carcinoma
122	49	F	breast carcinoma	166	71	M	non-small-cell lung carcinoma
123	46	F	breast carcinoma	167	61	M	non-small-cell lung carcinoma
124	39	F	breast carcinoma	168	52	F	non-small-cell lung carcinoma
125	42	F	breast carcinoma	169	72	M	non-small-cell lung carcinoma
126	64	F	breast carcinoma	170	36	F	non-small-cell lung carcinoma
127	34	F	breast carcinoma	171	59	F	non-small-cell lung carcinoma
128	35	F	breast carcinoma	172	51	F	non-small-cell lung carcinoma

173	67	M	non-small-cell lung carcinoma	207	72	M	hepatocirrhosis
174	60	M	non-small-cell lung carcinoma	208	58	M	hepatocirrhosis
175	55	M	non-small-cell lung carcinoma	209	67	M	hepatocirrhosis
176	45	M	non-small-cell lung carcinoma	210	88	M	hepatocirrhosis
177	82	F	non-small-cell lung carcinoma	211	71	M	hepatocirrhosis
178	59	F	non-small-cell lung carcinoma	212	67	M	hepatocirrhosis
179	71	M	non-small-cell lung carcinoma	213	53	M	hepatocirrhosis
180	79	M	non-small-cell lung carcinoma	214	72	F	hepatocirrhosis
181	66	M	non-small-cell lung carcinoma	215	38	M	hepatocirrhosis
182	47	M	non-small-cell lung carcinoma	216	32	M	hepatitis
183	26	F	non-small-cell lung carcinoma	217	41	M	hepatitis
184	32	F	non-small-cell lung carcinoma	218	66	F	hepatitis
185	33	M	non-small-cell lung carcinoma	219	47	F	hepatitis
186	49	M	non-small-cell lung carcinoma	220	28	F	hepatitis
187	56	F	non-small-cell lung carcinoma	221	29	M	hepatitis
188	32	M	non-small-cell lung carcinoma	222	66	F	hepatitis
189	39	F	non-small-cell lung carcinoma	223	38	M	hepatitis
190	45	M	non-small-cell lung carcinoma	224	21	M	hepatitis
191	66	M	non-small-cell lung carcinoma	225	31	F	hepatitis
192	58	M	non-small-cell lung carcinoma	226	15	F	hepatitis
193	64	F	hepatocirrhosis	227	38	M	hepatitis
194	51	F	hepatocirrhosis	228	51	F	hepatitis
195	57	F	hepatocirrhosis	229	70	M	hepatitis
196	76	M	hepatocirrhosis	230	29	M	hepatitis
197	44	F	hepatocirrhosis	231	44	M	hepatitis
198	53	F	hepatocirrhosis	232	53	F	hepatitis
199	61	M	hepatocirrhosis	233	55	M	hepatitis
200	68	M	hepatocirrhosis	234	37	M	hepatitis
201	81	F	hepatocirrhosis	235	39	M	hepatitis
202	58	F	hepatocirrhosis	236	59	M	hepatitis
203	75	F	hepatocirrhosis	237	35	M	hepatitis
204	56	M	hepatocirrhosis	238	59	F	hepatitis
205	67	M	hepatocirrhosis	239	32	M	hepatitis
206	22	F	hepatocirrhosis				

Table S4. Clinical features of 20 PC patients.

Case No.	Age	Gender	TNM stage	Cancer subtype
1	64	M	IV	pancreatic ductal adenocarcinoma
2	51	M	II	pancreatic ductal adenocarcinoma
3	66	M	III	pancreatic ductal adenocarcinoma
4	73	M	II	pancreatic ductal adenocarcinoma
5	64	M	III	pancreatic ductal adenocarcinoma
6	65	M	II	pancreatic ductal adenocarcinoma
7	63	F	IV	pancreatic ductal adenocarcinoma
8	52	M	IV	pancreatic ductal adenocarcinoma
9	44	F	III	pancreatic ductal adenocarcinoma
10	53	F	III	pancreatic ductal adenocarcinoma
11	54	M	IV	pancreatic ductal adenocarcinoma
12	72	M	II	pancreatic ductal adenocarcinoma
13	71	M	III	pancreatic ductal adenocarcinoma
14	65	M	II	pancreatic ductal adenocarcinoma
15	73	F	IV	pancreatic ductal adenocarcinoma
16	66	M	II	pancreatic ductal adenocarcinoma
17	59	M	III	pancreatic ductal adenocarcinoma
18	77	M	III	pancreatic ductal adenocarcinoma
19	76	F	II	pancreatic ductal adenocarcinoma
20	56	M	III	pancreatic ductal adenocarcinoma

Table S5. Clinicopathologic characteristics of tRF-Pro-AGG-004 and tRF-Leu-CAG-002 expression in cohort 1 (60 PC patients).

		**Tissue tRF-Pro-AGG-004**		**Tissue tRF-Leu-CAG-002**	
**Characteristics**	**n**	**ISH score (Mean±SD)**	***P* Value**	**ISH score (Mean±SD)**	***P* Value**
**Sex**			0.3835		0.576
Male	35	2.17±1.06		2.57±1.1	
Female	25	2.44±1.1		2.4 ± 1.09	
**Age(years)**			0.9129		0.8256
<Median(66.5)	30	2.3±1.08		2.53±1.08	
≥Median(66.5)	30	2.27±1.08		2.47±1.08	

Table S6. Clinicopathologic characteristics of tRF-Pro-AGG-004 and tRF-Leu-CAG-002 expression in cohort 2 (75 PC patients).

		**Tissue tRF-Pro-AGG-004**		**Tissue tRF-Leu-CAG-002**	
**Characteristics**	**n**	**ISH score (Mean±SD)**	***P* Value**	**ISH score (Mean±SD)**	***P* Value**
**Sex**			0.1218		0.9158
Male	49	2.36±1.16		3.23±0.91	
Female	26	2.81±1.23		3.21± 1.06	
**Age(years)**			0.2955		0.6837
<Median(62)	36	2.36±1.2		3.18±1.03	
≥Median(62)	39	2.65±1.19		3.27±0.9	

Table S7. tRF-Pro-AGG-004 pathway enrichment analysis.

**Pathwayenrichment analysis**				
**SourceDB**	**Term.id**	**Term.name**	**Intersection**	**P value**
Gene Ontology	GO:0048747	muscle fiber development	TTN/OBSL1/HDAC4/KLHL40/GPX1/HDAC9/FLNC/SMO/BIN3/NEBL/MYOM1/AFG3L2/MYBPC2	2.88E-07
Gene Ontology	GO:0055002	striated muscle cell development	OBSCN/ACTN2/TTN/OBSL1/HDAC4/KLHL40/GPX1/BVES/HDAC9/FLNC/SMO/BIN3/ADRA1A/NEBL/SIX4/ALPK3/MYH10/MYOM1/AFG3L2/MYBPC2	2.34E-06
Gene Ontology	GO:0050650	chondroitin sulfate proteoglycan biosynthetic process	B3GALT6/BCAN/B3GAT2/DSE/CHST12/CHST3/CHST9/NCAN	4.99E-06
Gene Ontology	GO:0055001	muscle cell development	OBSCN/ACTN2/TTN/OBSL1/HDAC4/KLHL40/GPX1/BVES/HDAC9/FLNC/SMO/BIN3/ADRA1A/NEBL/SIX4/ALPK3/MYH10/MYOM1/AFG3L2/MYBPC2	6.97E-06
Gene Ontology	GO:0050654	chondroitin sulfate proteoglycan metabolic process	B3GALT6/BCAN/ARSB/B3GAT2/DSE/CHST12/CHST3/CHST9/NCAN	1.12E-05
Gene Ontology	GO:0098742	cell-cell adhesion via plasma-membrane adhesion molecules	CNTN2/NECTIN3/CLSTN2/ADGRL3/PCDHGB1/PCDHGB2/PCDHGA7/CLDN12/PTPRD/NTNG2/FAT3/APOA1/DSCAML1/ROBO3/MAP2K5/CDH15/MAPK7/DSC1/MBP/ADGRL1/LRFN3/CDH22/CELSR1/PLXNB3	1.32E-05
Gene Ontology	GO:0030206	chondroitin sulfate biosynthetic process	B3GALT6/BCAN/DSE/CHST12/CHST3/CHST9/NCAN	1.52E-05
Gene Ontology	GO:0007266	Rho protein signal transduction	CNKSR1/ARHGEF2/OBSCN/ARHGEF4/FARP2/TRIO/COL1A2/ADRA1A/DNMBP/ARHGEF17/APOA1/SPATA13/SPATA13/MCF2L/RASGRF1/ARHGEF18/ARHGEF1/EPS8L1/CDC42EP1/CELSR1	3.16E-05
Gene Ontology	GO:0030204	chondroitin sulfate metabolic process	B3GALT6/BCAN/ARSB/DSE/CHST12/CHST3/CHST9/NCAN	3.53E-05
Gene Ontology	GO:0035023	regulation of Rho protein signal transduction	ARHGEF2/OBSCN/ARHGEF4/FARP2/TRIO/ADRA1A/DNMBP/ARHGEF17/APOA1/SPATA13/SPATA13/MCF2L/RASGRF1/ARHGEF18/ARHGEF1/EPS8L1	4.06E-05
KEGG	hsa04972	Pancreatic secretion	CELA3B/ATP1A2/TRPC1/PLA2G12A/ITPR3/ADCY1/CFTR/SLC4A2/RAP1B/PLCB2/ADCY7/ATP1A3/GNAS	8.48E-05
KEGG	hsa04934	Cushing syndrome	TCF7L1/TCF7/ITPR3/ADCY1/TCF7L2/KMT2A/RAP1B/PLCB2/USP8/ARMC5/ADCY7/AIPL1/DVL2/AXIN2/APC2/GNAS	0.000174
KEGG	hsa04724	Glutamatergic synapse	PPP3R1/GRM2/TRPC1/ITPR3/ADCY1/GRM3/GRK2/SLC38A1/PLCB2/JMJD7/PLA2G4B/GNB5/ADCY7/GNAS	0.000265
KEGG	hsa04726	Serotonergic synapse	TRPC1/KCNN2/DUSP1/ITPR3/KCND2/HTR5A/TPH1/KCNJ5/PLCB2/JMJD7/PLA2G4B/GNB5/GNAS/MAOB	0.000289
KEGG	hsa04390	Hippo signaling pathway	PRKCZ/TCF7L1/GLI2/WWTR1/DLG1/TCF7/PPP2CA/SCRIB/TCF7L2/TEAD4/DVL2/AXIN2/SMAD2/APC2/WTIP	0.000635
KEGG	hsa05165	Human papillomavirus infection	PRKCZ/COL9A2/TCF7L1/STAT1/DLG1/TCF7/PPP2CA/COL1A2/HEY1/SCRIB/IFNA13/IFNA1/TCF7L2/ATM/ITGA7/TRAF3/UBE3A/DVL2/AXIN2/SLC9A3R1/APC2/AKT2/GNAS/IKBKG	0.001049
KEGG	hsa05225	Hepatocellular carcinoma	TCF7L1/TCF7/ARID1B/TCF7L2/IGF2/IGF1R/DVL2/AXIN2/SMAD2/APC2/KEAP1/SMARCA4/DPF1/AKT2/TXNRD2	0.001278
KEGG	hsa04918	Thyroid hormone synthesis	ATP1A2/GPX1/ITPR3/ADCY1/GPX2/PLCB2/ADCY7/ATP1A3/GNAS	0.001467
KEGG	hsa04971	Gastric acid secretion	ATP1A2/ITPR3/ADCY1/CFTR/SLC4A2/PLCB2/ADCY7/ATP1A3/GNAS	0.001774
KEGG	hsa05217	Basal cell carcinoma	TCF7L1/GLI2/TCF7/SMO/TCF7L2/DVL2/AXIN2/APC2	0.002021

Table S8. tRF-Leu-CAG-002 pathway enrichment analysis.

**Pathwayenrichment analysis**				
**SourceDB**	**Term.id**	**Term.name**	**Intersection**	**P value**
Gene Ontology	GO:0072665	protein localization to vacuole	VPS13D/LAMTOR5/MON1A/SORL1/VPS4A/GGA3/TRAPPC8/CACNG7	4.64E-05
Gene Ontology	GO:0023061	signal release	KCNC4/ILDR2/SOX11/KLF7/PASK/WNT7A/CACNA2D2/CYB5R4/OPRM1/ADRA1A/NRXN2/PDX1/CYP19A1/EXOC3L1/SREBF1/GIT1/RASL10B/FBXL20/BRSK1/IL11/CHRNA4/PLA2G6/CDK16	0.000175
Gene Ontology	GO:1903522	regulation of blood circulation	GSTM2/CACNA1S/CACNA2D2/HRH2/PLN/ADRA1A/KCNE3/SCN4B/SCN3B/HCN4/ATP2A1/SREBF1/TBX2/ACE/SPTBN4/CACNG7/FGF13	0.000176
Gene Ontology	GO:0008016	regulation of heart contraction	GSTM2/CACNA1S/CACNA2D2/PLN/ADRA1A/KCNE3/SCN4B/SCN3B/HCN4/ATP2A1/SREBF1/TBX2/SPTBN4/CACNG7/FGF13	0.000227
Gene Ontology	GO:0051651	maintenance of location in cell	RER1/VPS13D/ARHGAP21/HK1/SORL1/ATP2A1/SPTBN4/TMSB15B	0.000556
Gene Ontology	GO:0060047	heart contraction	GSTM2/CACNA1S/CACNA2D2/PLN/ADRA1A/KCNE3/SCN4B/SCN3B/HCN4/ATP2A1/SREBF1/TBX2/ACE/SPTBN4/CACNG7	0.00074
Gene Ontology	GO:0003014	renal system process	CD34/HYAL2/AQP1/ADRA1A/KCNMA1/ADCY6/CORO2B/COMT/BCR	0.000867
Gene Ontology	GO:0061337	cardiac conduction	CACNA1S/CACNA2D2/PLN/KCNE3/SCN4B/SCN3B/HCN4/ATP2A1/SPTBN4/CACNG7	0.000918
Gene Ontology	GO:0061462	protein localization to lysosome	LAMTOR5/SORL1/VPS4A/GGA3/CACNG7	0.000981
Gene Ontology	GO:0003015	heart process	GSTM2/CACNA1S/CACNA2D2/PLN/ADRA1A/KCNE3/SCN4B/SCN3B/HCN4/ATP2A1/SREBF1/TBX2/ACE/SPTBN4/CACNG7	0.001041
KEGG	hsa04614	Renin-angiotensin system	PREP/MAS1/ACE	0.014548
KEGG	hsa00592	alpha-Linolenic acid metabolism	PLA2G4B/ACOX1/PLA2G6	0.018281
KEGG	hsa04514	Cell adhesion molecules (CAMs)	CD34/PDCD1/TIGIT/NRXN2/SPN/GLG1/ICOSLG/CLDN5	0.019145
KEGG	hsa04924	Renin secretion	CACNA1S/AQP1/KCNMA1/ADCY6/ACE	0.019815
KEGG	hsa04261	Adrenergic signaling in cardiomyocytes	CACNA1S/CACNA2D2/PLN/ADRA1A/SCN4B/ADCY6/ATP2A1/CACNG7	0.019853
KEGG	hsa05414	Dilated cardiomyopathy (DCM)	CACNA1S/CACNA2D2/PLN/ADCY6/ATP2A1/CACNG7	0.021596
KEGG	hsa00564	Glycerophospholipid metabolism	ACHE/PLPP5/PLA2G4B/PCYT2/PLD3/PLA2G6	0.023638
KEGG	hsa04270	Vascular smooth muscle contraction	CACNA1S/PPP1R12B/ADRA1A/KCNMA1/ADCY6/PLA2G4B/PLA2G6	0.030249
KEGG	hsa00480	Glutathione metabolism	GSTM4/GSTM2/GSTM5/IDH2	0.037677
KEGG	hsa05210	Colorectal cancer	RALB/RALA/TGFBR1/AXIN2/BBC3	0.045323
